# A simple method for low-contrast detectability, image quality and dose optimisation with CT iterative reconstruction algorithms and model observers

**DOI:** 10.1186/s41747-017-0023-4

**Published:** 2017-10-23

**Authors:** Luca Bellesi, Rolf Wyttenbach, Diego Gaudino, Paolo Colleoni, Francesco Pupillo, Mauro Carrara, Antonio Braghetti, Carla Puligheddu, Stefano Presilla

**Affiliations:** 1 Medical Physics Unit, Ente Ospedaliero Cantonale, Ospedale San Giovanni, Bellinzona, 6500 Switzerland; 2 Department of Radiology, Ente Ospedaliero Cantonale, Ospedale San Giovanni, Bellinzona, Switzerland; 3 University of Bern, Bern, Switzerland

**Keywords:** Low-contrast object detection, Computed tomography, Image quality, Model-based iterative reconstruction, Model observer

## Abstract

**Background:**

The aim of this work was to evaluate detection of low-contrast objects and image quality in computed tomography (CT) phantom images acquired at different tube loadings (i.e. mAs) and reconstructed with different algorithms, in order to find appropriate settings to reduce the dose to the patient without any image detriment.

**Methods:**

Images of supraslice low-contrast objects of a CT phantom were acquired using different mAs values. Images were reconstructed using filtered back projection (FBP), hybrid and iterative model-based methods. Image quality parameters were evaluated in terms of modulation transfer function; noise, and uniformity using two software resources. For the definition of low-contrast detectability, studies based on both human (i.e. four-alternative forced-choice test) and model observers were performed across the various images.

**Results:**

Compared to FBP, image quality parameters were improved by using iterative reconstruction (IR) algorithms. In particular, IR model-based methods provided a 60% noise reduction and a 70% dose reduction, preserving image quality and low-contrast detectability for human radiological evaluation. According to the model observer, the diameters of the minimum detectable detail were around 2 mm (up to 100 mAs). Below 100 mAs, the model observer was unable to provide a result.

**Conclusion:**

IR methods improve CT protocol quality, providing a potential dose reduction while maintaining a good image detectability. Model observer can in principle be useful to assist human performance in CT low-contrast detection tasks and in dose optimisation.

## Key points

- Detection of low-contrast objects and image quality in CT phantom images were evaluated
- Different tube loadings and image reconstruction methods were tested
- Iterative reconstruction in CT provided significant mAs reduction without image detriment
- Model observers are useful for parameter optimisation in CT dose reduction tasks

## Background

The overall per caput mean effective dose per year to the population in European countries, due to X-ray procedures, is about 1.05 mSv. Computed tomography (CT), which is a key medical imaging modality within clinical diagnostic applications, contributes, on average, to 57% of this dose (range 5.31–83.1%) [1], with a mean value of 7.44 mSv [2]. In Switzerland, through 2013 the number of CT exams was 117 per 1000 inhabitants, with an average dose per exam of 8.54 mSv. CT alone contributed to about 70% of the collective dose, with an average annual effective dose of 1 mSv per inhabitant [3]. In light of these data, reduction of radiation dose from CT has become an essential field of study. In the last years, the advent of faster microprocessors, CT iterative reconstruction (IR) methods were launched to integrate already existing algorithms such as filtered back projection (FBP) as a way to reduce patient radiation exposure while maintaining high-contrast spatial resolution.

iDose4 (Philips iDose4™ system, Philips Healthcare, Cleveland, OH, USA) belongs to the first generation of iterative hybrid reconstruction algorithms, which combine FBP and IR algorithms [4]. On the contrary, Iterative Model Reconstruction (IMR; Philips Healthcare, Cleveland, OH, USA) is an advanced knowledge-based algorithm that models the process of physical data acquisition through the iterative minimisation of the differences between image raw data and the estimated image [5]. IMR differs from FBP methods in that the reconstruction becomes an optimisation process that takes into account data statistics, image statistics, and system models. IMR levels differ by number of processing cycles which increase concurrently with increasing levels. The main difficulty is to preserve an adequate diagnostic image quality reducing exposure mAs values and therefore reducing the dose to the patient [6, 7].

Medical image quality assessment involves both a scientific and philosophical approach to define how ‘well’ specific information of interest is obtained from images. One method to define medical image quality is called ‘statistical task-based assessment approach’ and consists of the evaluation of the observer performance during tasks such as patient classification or estimation of volume and/or other characteristics of tumours. However, studies based on human observers are resource-demanding and involve a significant variability of intra-observer and interobserver performance. Being able to extract as much statistical information as possible from the available images, computational model observers can be used as convenient and objective surrogates of human beings to predict and/or define their expected performance [6, 8].

In medical imaging, model observers were developed to study how system parameters affect signal detection [9], taking into account physical factors that degrade image quality. They are also useful to evaluate and optimise software systems, such as image reconstruction or processing methods, both to study and predict their effects on human-observer performance [10–12].

The purpose of this work was to evaluate image quality and low-contrast object detectability in CT phantom images acquired at different tube loadings (i.e. mAs) and reconstructed with different algorithms in order to reduce mAs and consequently CT dose, with respect to a standard reference value, without detriment of the images. Model observer performance in terms of minimum diameter of the detectable low-contrast details was also evaluated.

## Methods

### CT phantom image acquisition

In this study, a Catphan 504 phantom (The Phantom Laboratory, Salem, NY, USA) was used to perform all image quality tests. It is a cylindrical phantom of 20-cm length and 20-cm diameter, containing several test modules: a solid image uniformity module (CTP486), a 21-line pair and point source high resolution module (CTP528), a module for slice width, sensitometry and pixel size evaluation (CTP401), and a low-contrast module (CTP515). In particular, the low contrast CTP515 module contains two sub-regions: the supraslice region with three groups of low-contrast objects, consisting of nine circular objects with diameters in the range of 2–15 mm and contrast of 0.3%, 0.5% and 1.0%, respectively, and a subslice region with three groups of four circular objects each (diameters in the range of 3–9 mm, contrast of 1.0%).

CT scans of the Catphan phantom were acquired with a Philips 256 iCT multi-slice CT unit (Brilliance iCT, Philips, Best, The Netherlands), aligning the phantom main axis with the axis of rotation of the scanner (z-axis). Acquisitions were performed selecting a beam collimation of 128 × 0.625 mm, scanning field of view of 214 mm, scan length of 213 mm, helical acquisition with 0.976 pitch factor, tube voltage of 100 kVp, scan time 2.5 s, rotation time 0.5 s, slice thickness 3 mm and zoom 100%.

The product of tube current and exposure time per rotation (i.e. tube load) was in the range of 15–300 mAs. Image reconstruction was performed using the following reconstruction algorithms: FBP; iDose with levels in the range of 1–6; and IMR with levels in the range of 1–3.

### Software for image analysis

For image quality analysis, one software product was used and compared, in terms of numerical results, with an advanced automated quality assurance software service available on the web. For the definition of low-contrast detectability, however, studies based on both human and model observers were performed.

CT image quality parameters were evaluated with two different software resources, CTQA_cp and Catphan QA (Image Owl, Inc., Greenwich, NY, USA), in order to cross-check the obtained results and validate CTQA_cp results with a reference. CTQA_cp (version 0.3.1) is a freeware software package developed to aid CT quality assurance programs and able to automatically produce image quality reports. In particular, the following parameters are analysed with CTQA_cp: slice thickness, pixel size, CT number linearity, uniformity, homogeneity, image noise across detector rows, and modulation transfer function (MTF). A low-contrast resolution analysis tool of the Catphan CP515 module based on a model observer is also available.

Catphan QA executes an automatic analysis of CT Catphan images and produces an image quality report. The following CT imaging performance parameters are evaluated: sensitometry; MTF (i.e. from beads and wires analysis); critical frequency; CT linearity; phantom position; rotation and yaw; slice width; and contrast detectability.

Catphan QA also includes a contrast diameter detail function that returns dimensions of the smallest detectable target for each of the three contrast values and was used in order to obtain image quality low-contrast information.

Image quality parameters were evaluated with CTQA_cp and Catphan QA and on the phantom images acquired with the different CT mAs values and reconstructed with different reconstruction algorithms.

### Physical metrics quantification with CTQA_cp and Catphan QA

For each adopted scanning protocol (i.e. different mAs) and reconstruction algorithms, noise, uniformity, and high-contrast spatial resolution were evaluated in order to quantify how the different CT acquisition parameters impact on the physical metrics. Both CTQA_cp and Catphan QA were used and the obtained results were compared.

#### Noise

Noise was characterised on the images of the Catphan CTP 485 uniform module as the standard deviation of pixel values within a square region of interest (ROI) located at the centre of the phantom module.

#### Uniformity

Uniformity was calculated in the homogeneous region of the CP486 module as the deviation in CT numbers of the mean value of upper, right, lower, and left circular off-centre ROIs from the mean value of a ROI placed at the centre of the image of the phantom. Position and dimension of the ROIs could change between the two software products. In any case, the closer to unity was the result, the more uniform was the image.

#### High-contrast spatial resolution

MTF was calculated as the Fourier transform of the point spread function of a region of interest centred on the lower bead point object of the Catphan CTP 528-point source module.

### Low-contrast spatial resolution

As described below, empirical and computational methods were evaluated in this study to quantify low-contrast spatial resolution.

#### Evaluation with the four-alternative forced test

Four-alternative forced-choice (4-AFC) [13] test was executed to evaluate low-contrast spatial resolution by five radiologists with at least 15 years of experience in clinical CT and four experienced radiology technicians [14]. Observers were trained on all technical aspects and objectives of the study and frontal training was performed through examples before the test.

**Fig. 1:**
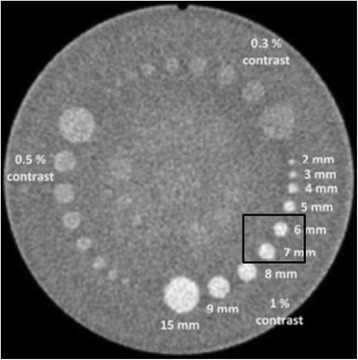
Image of the low-contrast module of the Catphan phantom

**Fig. 2:**
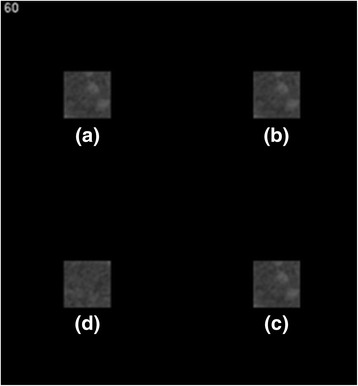
Example of image for the 4-AFC test. In this case, low-contrast objects were in quadrants **a**, **b** and **c** while quadrant **d** is empty

**Fig. 3:**
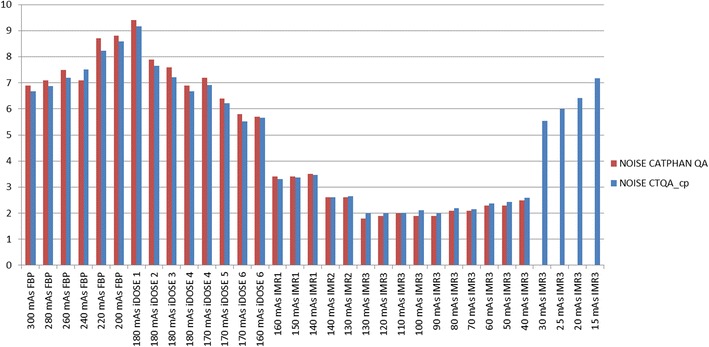
Noise quantification with Catphan QA and CTQA_cp for the different CT protocols and reconstruction algorithms

The 4-AFC test was performed in a darkened room with a constant level of low ambient lighting and images were presented on a DICOM-calibrated megapixel colour LCD screen (Radiforce RX320 LCD, EIZO Corporation) with a native resolution of 1536 × 2048. Initial window and level values of 100 and 1090 were suggested, respectively, but observers were free to modify them if necessary. No limitations on viewing distance and time were set and no reference image was provided before the start. Each human observer analysed 543 stacks of four images containing either just background or the 6-mm and 7-mm diameter objects (1% contrast) of the low-contrast supraslice region of the Catphan phantom. To create the stack of images for the 4-AFC test, dedicated macros were created using the freeware software ImageJ (National Institute of Health Image, Bethesda, MD, USA) that automatically executes the following steps: (1) extracts samples of the low-contrast objects (diameters 6–7 mm, 1% contrast) or of the background from low-contrast Catphan module (Fig. 1); (2) generates a series of images each containing four quadrants with low-contrast circular objects or background, randomly chosen from Catphan images acquired at different experimental conditions (i.e. mAs in the range of 30–300) and reconstructed by means of FBP, iDOSE (i.e. levels 1–6) and IMR (i.e. levels 1–3). Sixteen images for each CT protocol modality were overall selected and randomly arranged over the stack of 543 images. One example of images is provided in Fig. 2, each quadrant possibly representing the particular of the Catphan CTP515 low-contrast module shown in Fig. 1; (3) creates a stack of 543 images in a single DICOM image sequence that was loaded to a picture archiving and communication system PACS (Philips IntelliSpace PACS Enterprise 4.4.532.1, Philips Healthcare Informatics, Inc., Foster City, CA, USA) for further evaluation by the observers.

In this study, observers had to identify the presence of one or more quadrants with low-contrast lesions and to indicate their position within the image. In principle, in each one of the 543 images, low-contrast objects were in none, one, two, three, or any quadrant. The percentage of correct answers given by each observer subjected to the 4-AFC experiment was analysed and evaluated. Inter-CT protocol modality (i.e. each combination of mAs and reconstruction algorithms) analysis was performed.

#### Computational evaluation

The computer model observer provided with CTQA_cp was used to define low-contrast detectability on the Catphan low-contrast supraslice images acquired at different experimental conditions (i.e. mAs in the range of 15–300) and reconstructed by means of FBP, iDOSE (i.e. levels 1–6) and IMR (i.e. levels 1–3). According to the method, which is exhaustively described by Hernandez-Giron et al. [6], output of the software system is the smallest ‘visible’ object size at 1%, 0.5%, and 0.1% contrast. Only objects with 1% contrast were evaluated because 0.5% and 0.1% objects were often not visible during first visual evaluations after phantom CT acquisitions. Catphan QA also includes a function for low-contrast diameter detail evaluation, which returns dimensions of the smallest detectable target for each of the three contrast values. This function is not based on a model observer-based statistical approach, but it is related on the use of an algorithm for image analysis.

## Results

### Physical metrics quantification with CTQA_cp and Catphan QA

**Fig. 4:**
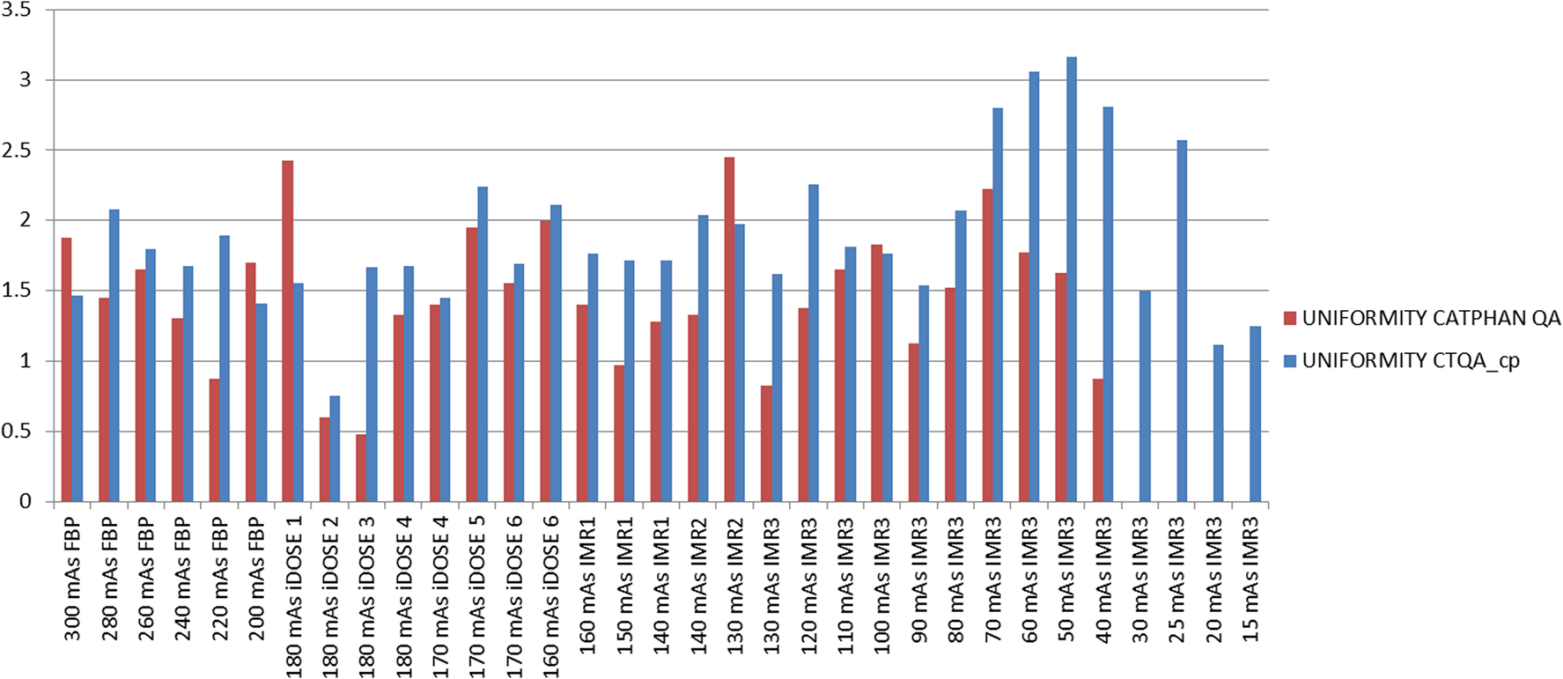
Uniformity quantification with Catphan QA and CTQA_cp for the different CT protocols and reconstruction algorithms

Noise and uniformity evaluations are provided in Figs. 3 and 4, respectively; the high-contrast spatial resolutions for 50% and 10% MTF are given in Table 1. Numerical results of both software systems resulted to be comparable in terms of noise analysis, whereas a difference arose for uniformity. High-contrast spatial resolutions evaluated with Catphan QA resulted furthermore systematically higher than those evaluated with CTQA_cp, although the difference was limited and always below 1. At mAs values less than or equal to 30, Catphan QA was unable to quantify MTF. Uniformity results, shown in Fig. 4, showed small deviations variability, especially below 80 mAs, for CTQA_cp.

**Table 1:** High-contrast resolution results for 50% and 10% MTF, obtained with Catphan QA and CTQA_cp

MTF results	Catphan QA		CTQA_cp	
CT acquisition parameter	MTF (ll/cm) 50%	MTF (ll/cm) 10%	MTF (ll/cm) 50%	MTF (ll/cm) 10%
300 mAs FBP	3.7	6.4	3.0	6.1
280 mAs FBP	3.7	6.3	3.0	6.5
260 mAs FBP	3.7	6.4	3.5	6.1
240 mAs FBP	3.7	6.3	3.0	6.1
220 mAs FBP	3.8	6.5	3.5	6.5
200 mAs FBP	3.9	6.6	3.1	6.5
180 mAs IDOSE 1	3.8	6.5	2.8	6.0
180 mAs IDOSE 2	3.7	6.5	3.1	6.0
180 mAs IDOSE 3	3.8	6.5	3.5	6.5
180 mAs IDOSE 4	3.9	6.6	3.4	6.0
170 mAs IDOSE 4	3.8	6.5	3.0	6.0
170 mAs IDOSE 5	3.8	6.5	3.0	6.1
170 mAs IDOSE 6	3.9	6.7	3.0	6.1
160 mAs IDOSE 6	3.7	6.5	2.9	5.6
160 mAs IMR1	4.2	7.1	3.6	6.5
150 mAs IMR1	4.1	7.1	3.6	6.6
140 mAs IMR1	4.1	6.9	3.5	6.5
140 mAs IMR2	4.0	6.8	3.7	6.4
130 mAs IMR2	4.2	7.1	3.8	6.5
130 mAs IMR3	4.0	6.8	3.5	6.3
120 mAs IMR3	3.9	6.7	3.5	6.0
110 mAs IMR3	4.1	7.0	3.9	6.5
100 mAs IMR3	3.9	6.7	3.5	6.1
90 mAs IMR3	3.9	6.7	3.6	6.1
80 mAs IMR3	4.0	6.8	3.6	6.4
70 mAs IMR3	3.6	6.2	3.5	6.0
60 mAs IMR3	3.8	6.5	3.5	5.9
50 mAs IMR3	3.4	6.0	3.6	5.7
40 mAs IMR3	3.4	6.0	3.2	5.6
30 mAs IMR3	NE	NE	4.4	6.9
25 mAs IMR3	NE	NE	3.5	6.0
20 mAs IMR3	NE	NE	3.7	7.0
15 mAs IMR3	NE	NE	4.0	7.0

*NE* not evaluated by the software

### Low-contrast spatial resolution evaluation

**Fig. 5:**
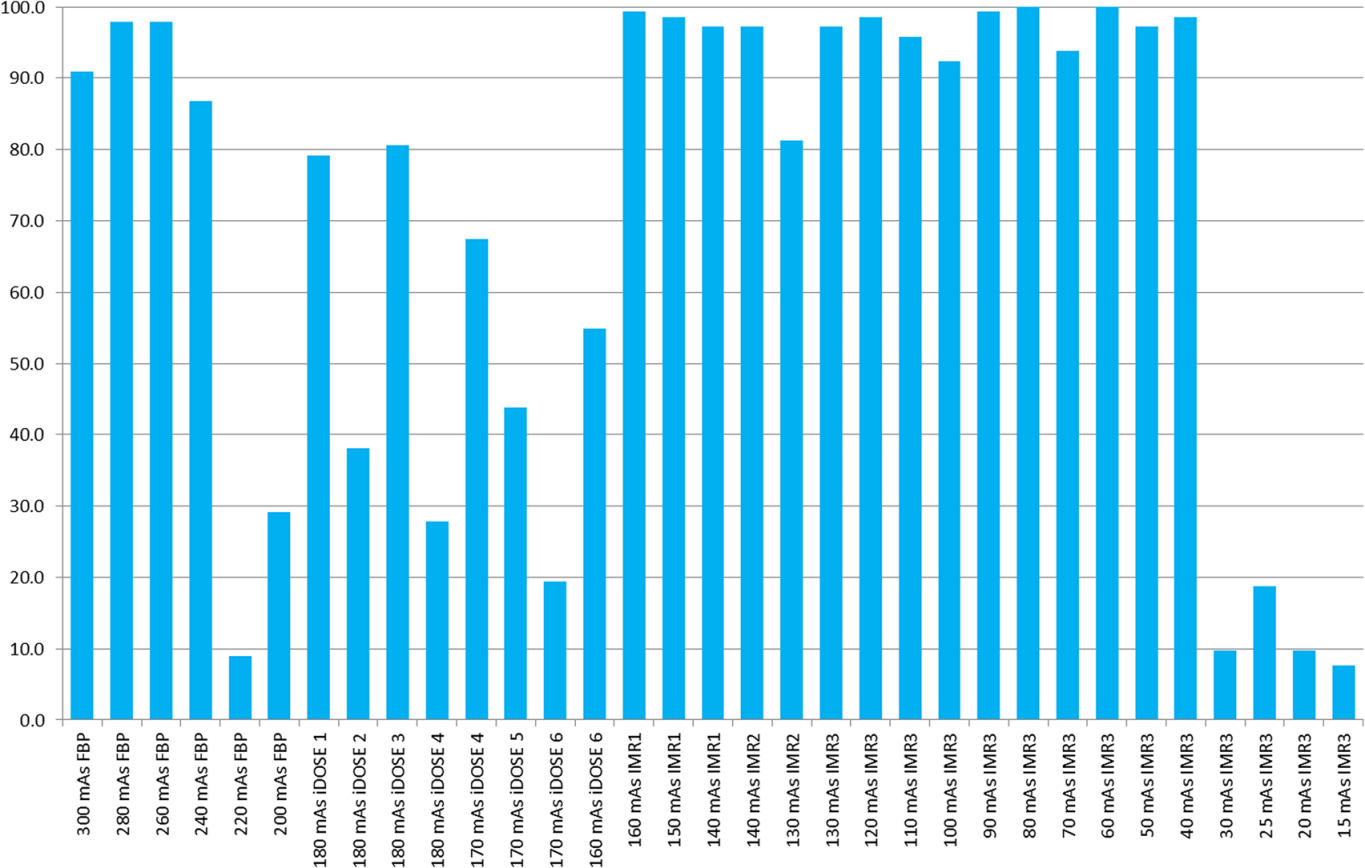
Percentage of mean correct answers (*histogram*) and of their variability (*error bars* representing the standard deviation, k = 1) at changing CT protocols and reconstruction algorithms

Figure 5 shows the average and standard deviation of the percentage of correct answers provided by the human observers at changing CT protocol. For mAs in the range from 240 to 160, using FBP or iDOSE (levels 1–6), the average of correct answers is suboptimal, indicating a net degradation of the perceived image quality and of the low-contrast object detectability [15]. Introducing IMR (levels 1–3), the average of the percentage of correct answers increases significantly and remains above 90% while lowering the mAs values up to 40.

Table 2 shows Catphan QA low-contrast results. Detectability of objects with 1% contrast is incremented from 3 mm to 2 mm details with the introduction of IMR, indicating that the use of iterative algorithms slightly improves the detection of low-contrast objects [16].

**Table 2:** 1%, 05% and 0.3% low-contrast detectability obtained with Catphan QA

CT acquisition parameter	Detail at 1% contrast (mm)	Detail at 0.5% contrast (mm)	Detail at 0.3% contrast (mm)
300 mAs Standard	2	5	7
280 mAs Standard	3	5	7
260 mAs Standard	3	5	7
240 mAs Standard	3	5	7
220 mAs Standard	3	5	8
200 mAs Standard	3	6	8
180 mAs ISODOSE 1	3	6	9
180 mAs ISODOSE 2	3	6	9
180 mAs ISODOSE 3	3	5	9
180 mAs ISODOSE 4	3	5	8
170 mAs ISODOSE 4	3	6	9
170 mAs ISODOSE 5	3	5	8
170 mAs ISODOSE 6	2	5	9
160 mAs ISODOSE 6	3	5	9
160 mAs IMR1	2	5	6
150 mAs IMR1	2	3	6
140 mAs IMR1	2	3	6
140 mAs IMR2	2	3	5
130 mAs IMR2	2	3	6
130 mAs IMR3	2	2	5
120 mAs IMR3	2	3	6
110 mAs IMR3	2	3	6
100 mAs IMR3	2	3	6
90 mAs IMR3	2	2	5
80 mAs IMR3	2	3	7
70 mAs IMR3	2	3	6
60 mAs IMR3	2	3	6
50 mAs IMR3	2	3	8
40 mAs IMR3	2	4	15
30 mAs IMR3	NE	NE	NE
25 mAs IMR3	NE	NE	NE
20 mAs IMR3	NE	NE	NE
15 mAs IMR3	NE	NE	NE

*NE* not evaluated

Table 3 shows model observer results in terms of minimum diameter of the detectable low-contrast details. Results from 300 to 200 mAs showed a high variability, whereas from 180 to 100 mAs they were almost constant, with the diameters of the minimum detectable detail all being around 2 mm. Below 100 mAs the software system was unable to detect objects probably due to intrinsic algorithm limitations.

**Table 3:** 1% low-contrast detectability obtained with the model observer in CTQA_cp

CT acquisition parameter	Detail at 1% contrast (mm)
300 mAs Standard	5.8
280 mAs Standard	7.6
260 mAs Standard	15
240 mAs Standard	0
220 mAs Standard	2
200 mAs Standard	2
180 mAs ISODOSE 1	0
180 mAs ISODOSE 2	2
180 mAs ISODOSE 3	2.2
180 mAs ISODOSE 4	2
170 mAs ISODOSE 4	2.4
170 mAs ISODOSE 5	2.3
170 mAs ISODOSE 6	2
160 mAs ISODOSE 6	2.1
160 mAs IMR1	2
150 mAs IMR1	2
140 mAs IMR1	2.5
140 mAs IMR2	2
130 mAs IMR2	2
130 mAs IMR3	2
120 mAs IMR3	2
110 mAs IMR3	2
100 mAs IMR3	2
90 mAs IMR3	ND
80 mAs IMR3	ND
70 mAs IMR3	ND
60 mAs IMR3	ND
50 mAs IMR3	ND
40 mAs IMR3	ND
30 mAs IMR3	ND
25 mAs IMR3	ND
20 mAs IMR3	ND
15 mAs IMR3	ND

*ND* not detected by the software

## Discussion

The purpose of this work was to use IR algorithms for obtaining a percentage threshold value of mAs in order to reduce CT dose while maintaining image quality. Human and computational detection performances were also evaluated. In general, results obtained by means of CTQA_cp and Catphan QA in terms of image quality were approximately in agreement. The resolution of a CT imaging system is well characterised with the MTF, which indicates its ability to reproduce various levels of detail from a region of the patient to its image. Small differences obtained for uniformity and MTF are likely due to small differences between the applied calculation algorithms. In particular, for uniformity analysis, position and dimension of the ROIs may change between CTQA_cp and Catphan QA. It is only specified that in CTQA_cp the area of the ROIs correspond to the area of a circle with diameter 10% of the diameter of the homogeneous region in the CP486 module. Whereas for Catphan QA, it is indicated that the outer edge of each ROI is located 1 cm from module border. For MTF evaluation, the small changes might be due to differences in the Fourier transform analysis of the images.

Image quality analysis anyway confirmed data already reported in literature, supporting the efficiency of the novel IR methods if compared to standard reconstruction algorithms such as FBP [17, 18]. Regarding noise, it initially increased while mAs values were lowered using FBP reconstruction. The application of iDose constantly reduced it, even at decreasing mAs, and IMR kept it low while tube loading reduced to 50 mAs. Below this value, noise increased with a consequent degradation of the image quality due to IR limits at very low mAs values [19]. It was observed that the uniformity values were within the advised limit (ΔHU ≤ 4) [20]. iDOSE provided, therefore, a similar image resolution to that obtained with FBP at significantly higher mAs values. Our results represent, therefore, a valuable confirmation that the use of IR algorithms preserves the spatial resolution while reducing mAs [21], except at very low tube load (i.e. < 50 mAs) where a very small decrease in spatial resolution was found [19].

Evaluation of the low-contrast performance in CT imaging is a difficult task. It is related to the ability of an operator to distinguish between two objects or regions with similar CT number and it depends on statistical noise levels, contrast and size of the signal.

Referring to Fig. 5, on the one hand the percentage of correct answers is a proper quantification of the efficiency of the application of the various reconstruction algorithms for low-contrast details identification, on the other hand the standard deviation is a good descriptor of inter-observer’s variability of image quality evaluation. The comparison between the different acquisition and image reconstruction modalities confirmed the highest efficiency for IMR, level 3 [22]. In fact, in all tested conditions low-contrast detection rates were greater for IMR than for FBP or iDOSE; low-contrast detectability was preserved with IMR up to a tube loading reduction to 40 mAs. In accordance to Katsura et al. [23], a consequent decrease of dose to the patient by a factor up to 7 (i.e. 80% dose reduction) seems, therefore, to be possible without producing any significant detriment to the images.

Interestingly, the variability of the percentage of correct answers was high for iDose in the range of 220–160 mAs, whereas it was much lower with IMR, even at reduced tube loading. This was due to possible reconstruction limitations of iDOSE, which might stress the perception variability among observers. As previously described, psychological factors could, in fact, affect the test results [10]. As observers have performed the test in different moments of the day, diagnostic accuracy, visual accommodation, reading time, subjective ratings of fatigue and visual strain, before and after a day of clinical reading, may all have contributed as confounding factors in terms of image quality evaluations. Image texture, artefacts and over-smoothing of images with higher strengths of IR may have affected diagnostic results [24].

One could argue that, different from our work, a large amount of papers compared iterative with exact methods for specific clinical applications (e.g. morphological evaluation of tumours) in specific anatomical regions and diseases. We did not focus on specific anatomical regions and diseases because the approach described in this study could be adopted in many different clinical applications, including the low-contrast regions/tissues detection task, such as liver lesion identification in abdominal CT [24, 25].

The performance of model observer software was, in general, good in terms of low-contrast detectability up to 100 mAs. Below this value, the model observer did not work well probably due to software intrinsic limitations. Different from human observers, model observer software recognised objects of 2 mm in diameter as a prediction of human observer performance also between 220 and 160 mAs. This difference was particularly evident on the 220-mAs images, where the mean value of correct answers by human observers was 9%, whereas according to the model observer a 2-mm diameter object is detectable.

The model observer given in CTQA_cp showed to be a valid tool for a first evaluation of the analysed data, but presented the following limitations that would require upgrades and improvements: (1) no optimisation/adaptation is possible to ‘instruct’ the system for specific study conditions; (2) no univocal and absolute detectability scoring is provided as output. In particular, a detectability scoring could be important to better quantify the right mAs reduction percentage, optimised in terms of human-perceived image quality. In general, a better model observer software, with sophisticated interfaces and specific setup possibilities, should probably be adopted in future to assist better and predict human observer’s performance. Implementation of a more advanced software, which is beyond the aim of this study, is, however, very complex, as it requires a thorough knowledge of model observer theory, statistics and informatics [26].

In conclusion, this study demonstrated that the application of the IR algorithm IMR to phantom images preserves a good image quality and object detectability for human radiological evaluation of CT exams, with a potential noise reduction up to 60% and, in particular, an 85% dose reduction to the patient. With respect to other studies, the method presented in this work can be easily implemented and contains a thorough analysis for the evaluation and optimisation of mAs according to the adopted reconstruction algorithms. The model observer can, in principle, be useful to assist human performance in CT low-contrast detection tasks and in dose optimisation, but needs to be optimised in order to extract useful information to support and predict human observer evaluations on CT images. Further studies are required to confirm the reported findings.

## Abbreviations

4-AFC: Four-alternative forced-choice
CT: Computed tomography
FBP: Filtered back projection
IMR: Iterative model reconstruction
MTF: Modulation transfer function
RA: Reconstruction algorithm
ROI: Region of interest
